# The potential cost-effectiveness of novel cord blood therapies in children with autism spectrum disorder

**DOI:** 10.1371/journal.pone.0282906

**Published:** 2023-04-18

**Authors:** Ethan D. Borre, Evan Myers, Marianne Hamilton Lopez, Joanne Kurtzberg, Beth Shaz, Jesse Troy, Gillian D. Sanders Schmidler

**Affiliations:** 1 Department of Population Health Sciences, Duke University School of Medicine, Durham, NC, United States of America; 2 Duke-Margolis Center for Health Policy, Duke University, Durham, NC, United States of America; 3 Division of Women’s Community and Population Health, Department of Obstetrics & Gynecology, Duke University School of Medicine, Durham, NC, United States of America; 4 Marcus Center for Cellular Cures, Duke University Medical Center, Durham, NC, United States of America; 5 Department of Biostatistics and Bioinformatics, Duke University School of Medicine, Durham, NC, United States of America; 6 Duke University Clinical Research Institute, Duke University School of Medicine, Durham, NC, United States of America; The University of Texas MD Anderson Cancer Center, UNITED STATES

## Abstract

**Objective:**

To model the long-term clinical and economic outcomes of potential cord blood therapy in autism spectrum disorder (ASD).

**Study design:**

Markov microsimulation of ASD over the lifespan was used to compare two strategies: 1) standard of care (SOC), including behavioral and educational interventions, and 2) novel cord blood (CB) intervention in addition to SOC. Input data reflecting behavioral outcomes included baseline Vineland Adaptive Behavior Scale (VABS-3), monthly VABS-3 changes, and CB intervention efficacy on adaptive behavior based on a randomized, placebo-controlled trial (DukeACT). Quality-adjusted life-years (QALYs) were correlated to VABS-3. Costs for children with ASD ($15,791, ages 2–17 years) and adults with ASD ($56,559, ages 18+ years), and the CB intervention (range $15,000–45,000) were incorporated. Alternative CB efficacy and costs were explored.

**Results:**

We compared model-projected results to published data on life-expectancy, mean VABS-3 changes, and lifetime costs. Undiscounted lifetime QALYs in the SOC and CB strategies were 40.75 and 40.91. Discounted lifetime costs in the SOC strategy were $1,014,000, and for CB ranged from $1,021,000-$1,058,000 with CB intervention cost ($8,000-$45,000). At $15,000 cost, CB was borderline cost-effective (ICER = $105,000/QALY). In one-way sensitivity analysis, CB cost and efficacy were the most influential parameters on CB ICER. CB intervention was cost-effective at costs<$15,000 and efficacies ≥2.0. Five-year healthcare payer projected budgetary outlays at a $15,000 CB cost were $3.847B.

**Conclusions:**

A modestly effective intervention designed to improve adaptive behavior in autism can be cost-effective under certain circumstances. Intervention cost and efficacy most affected the cost-effectiveness results and should be targeted to increase economic efficiency.

## 1. Introduction

Autism spectrum disorder (ASD) affects 1 in 54 children in US and has profound effects on self-reported quality of life (QOL) as well as QOL of family members [1, 2]. Further, medical care, social services, and lost productivity can cost up to $1.5 million over a lifetime [3]. Currently, there are no FDA-approved pharmacologic or medical treatments that target the core symptoms of autism. The current standard of care (SOC) treatment includes behavioral interventions that are costly, requiring up to 20 hours per week of expert time. Although randomized controlled trials have shown improvements in language and cognition associated with early intervention, many individuals with ASD continue to have significant impairments throughout their lifetime [4, 5].

Recently, two trials of cord blood infusion in children with ASD have shown safety and feasibility of the intervention [6, 7]. While a subsequent randomized trial (NCT02847182) did not show significant benefit of cord blood therapy on primary trial outcomes, post-hoc subgroup analyses suggested improvements in an adaptive behavior subdomain, as well as improvements in brain activity, as assessed via EEG, and in sustained attention, as assessed via eye-tracking measures [8]. There is large uncertainty around the potential benefits of cord blood therapy in ASD given its high upfront costs, and indeed if there is any benefit at all.

Our objective was to develop a decision modeling framework to assess the cost-effectiveness of potentially efficacious therapies for ASD to inform future clinical trials and future drug development. While the cord blood intervention in a recent trial did not meet its primary or secondary endpoints, understanding combinations of ASD treatment efficacies and costs likely to be cost-effective for other interventions will prove critical. Findings from this analysis might inform future clinical trials and researchers on clinical endpoints, provide a framework for considering value-based payment for therapies proven efficacious, and identify remaining uncertainties requiring further data acquisition.

## 2. Methods

### 2.1 Analytic overview

We developed a Markov microsimulation model of autism over the lifespan to compare two strategies: 1) SOC, including standard behavioral and educational interventions, and 2) cord blood (CB) which included the above interventions as well as a cord blood infusion intervention [6]. To inform our basecase input parameters, we incorporated cohort characteristics from, and grounded our basecase cord blood efficacy assumptions on, the DukeACT (Efficacy of Intravenous Umbilical Cord Blood Infusion as Cell Therapy for Children With ASD) trial. We simulated a cohort with characteristics derived from participants with non-verbal intelligence quotient (NVIQ) ≥70 in DukeACT from age 2 throughout their lifetime. The model was compared to literature that reported VABS trajectories and lifetime costs. Clinical and cost outcomes are reported both undiscounted, as in the budget impact analysis (BIA), and discounted at 3%/year for the cost-effectiveness analysis. Our analysis was conducted from a modified societal perspective, and we include a healthcare payer perspective as a sensitivity analysis (Appendix 5 in S1 File) [9]. We considered an ICER<$100,000/QALY cost-effective by US standards [10]. Please see Appendix 4 in S1 File for a Consolidated Health Economic Evaluation Reporting (CHEERS) checklist.

### 2.2 Model structure

Our Markov microsimulation model of ASD was developed using TreeAge (Williamstown, MA). Simulated participants with ASD enter the model at age 2 (health state *child*, *ages 2–18*) and are followed monthly, accruing quality-adjusted life years (QALYs) and costs (**S1 Fig in S1 File**). ASD natural history is tracked using VABS-3 scores, initially drawn from a distribution seen in DukeACT and subsequently following developmental trajectories from the published literature [11, 12]. VABS-3 is assumed to vary from ages 2–7 years, after which the score is fixed due to an absence of data on VABS-3 changes in individuals with ASD and without ID after age 7 [12]. VABS-3 has been shown to grow more rapidly in early childhood, plateauing as an individual ages, and we included sensitivity analyses exploring different rates of VABS-3 growth [13–15]. At age 18, simulated participants transition to one of three health states based on their VABS-3: 1) VABS-3>85, 2) VABS-3 70–85, and 3) VABS-3<70. Cutoff values were chosen using the mean and standard deviation of VABS-3, where a mean of 100 is equated to a typically developing peer, with a standard deviation of 15 [11]. Simulated participants also experienced monthly age-, sex-, and autism-specific mortality.

Intervention

DukeACT compared cord blood infusion to placebo and did not find an effect of cord blood treatment on the primary outcome, Vineland Adaptive Behavior Scale-III (VABS-3) socialization subdomain score at 6 months, or any of the pre-specified secondary outcomes [8], including the VABS-3 composite score. However, post-hoc subgroup analysis of participants without intellectual disability (IQ ≥ 70) showed improvement at 6 months in the communication subdomain of VABS-3 in the treatment arm compared to the placebo arm among participants without intellectual disability. Given the lack of other informative data, to inform our basecase analysis, we incorporated 6-month data from DukeACT on the change in VABS-3 subscale scores seen in the placebo group and CB group as the modeled SOC and intervention, respectively. In this analysis, we assumed that clinicians, regulatory bodies, and other decision makers would not pursue implementation of a non-efficacious therapy. In the basecase, we incorporated a difference between the SOC and intervention arms in the communication subscale score that was uncovered in post-hoc subgroup analysis of a secondary outcome in DukeACT (VABS-3 communication subdomain scores). We acknowledge that in DukeACT, cord blood therapy may have had no effect and included this possibility in cost-effectiveness and budget impact analysis.

At model initiation, simulated participants are assigned a 6-month change in VABS-3 communication subscale score (SOC vs. CB), based on DukeACT findings. Simulated participants also draw 6-month changes in socialization and daily living subscale scores from the same distributions seen in DukeACT (no difference between SOC and intervention arms). As a conservative measure, we simulate the SOC group as receiving the full increase in VABS-3 subscale scores seen in DukeACT placebo group.

### 2.3 Model input data

#### Cohort characteristics

Cohort characteristics were derived from 6-month DukeACT trial data. Age at initiation for all participants was 2 years and 78.2% were male (**Table 1**). NVIQ at initiation for simulated participants was 88.1, and Autism Diagnostic Observation Schedule (ADOS) calibrated severity score was 16.7.

**Table 1:** Model input data.

Variable	Value	Reference
Age, years	2	DukeACT
Sex, % male	78.2	
Non-verbal IQ	88.1	
Autism Diagnostic Observation Schedule Calibrated Severity Score	16.7	
Baseline VABS-3 composite, mean (SD)	71.8 (11.8)	
Mean VABS-3 composite change ages 2-4y, monthly	0.05 (0.01)	[12]
Mean VABS-3 composite change ages 4-5y, monthly	0.14 (0.02)	
Mean VABS-3 composite change ages 5-6y, monthly	0.14 (0.02)	
Mean VABS-3 composite change ages 6-7y, monthly	0.14 (0.02)	
Intervention efficacy, 6-month mean (SD) change in VABS-3 communication subscale scores		DukeACT
SOC	0.1 (7.3)	
CB	3.0 (7.9)	
6-month mean (SD) change in VABS-3 socialization subscale score, both groups	3.2 (9.2)	DukeACT
6-month mean (SD) change in VABS-3 daily living subscale score, both groups	2.8 (6.7)	
CB Safety, % occurrence		
Mild/Moderate event	9.2%	DukeACT
Severe event	3.4%	
**Costs, 2019 USD**	**Value**	**Reference**
Yearly healthcare and societal costs, ages 2–17 years	19,199	[20]
Yearly healthcare costs, ages 2–17 years*	3,395	
Yearly costs, ages 18+		
Accommodation	20,322	[3]
Employment support	396	
Medical services*	15,264	
Nonmedical services	6,399	
Caregiver productivity loss	2,131	
Individual productivity loss	12,047	
Cord blood Infusion	15,000	[23–25]
CB Toxicity cost, severe	11,571	Assumption
Adult labor-force participation, full- or part-time employment, %		
VABS-3 composite >85	60	Assumption
VABS-3 composite 70–85	40	
VABS-3 composite <70	20	

CB: cord blood, IQ: intelligence quotient, SD: standard deviation, SOC: standard of care, VABS-3: Vineland Adaptive Behavior Scale-3.

*Included in the healthcare payer perspective

#### Autism natural history

Baseline VABS composite scores for each simulated person were drawn from a distribution (mean = 71.8, standard deviation = 11.8; Table 1) at model initiation. Monthly increases in VABS scores derived from a longitudinal natural history study from the National Institutes of Health inform the simulation of ASD developmental trajectory [12]. VABS changes from the final growth mixture model over each year of life in the higher developing group were assumed to occur linearly, converted to age-specific monthly changes, and applied between the simulated subscale scores (**Table 1**). We calculated age-, sex-, and autism-specific mortality by using US lifetables and standardized mortality ratios in persons with ASD and without intellectual disability [16, 17]. We used 2017 US lifetable age- and sex-specific yearly death probabilities, calculated monthly rates, and applied male and female SMRs from Hirvikoski et al. independently (2.49/1.88 for male/female), assuming the SMR for high-functioning autism applied across the entire lifespan [16]. We then combined male and female monthly rates using the percent male (78%), and converted back to probabilities. Due to an absence of data reporting the mean life expectancy of persons with ASD and without intellectual disability, we used this source and acknowledge that our SMRs may overestimate mortality in persons without intellectual disability.

#### Intervention efficacy

The mean 6-month change in VABS-3 communication subscale score in DukeACT for individuals with IQ≥70 receiving CB was 3.0 (SD = 7.9) (Appendix 2 in S1 File). Participants in the SOC group received the mean change of VABS-3 communication subscale score observed in the placebo group in DukeACT of 0.1 (SD = 7.3). Both the simulated SOC and intervention arms received 6-month mean changes in socialization and daily living subscale scores of 3.2 (SD = 9.2) and 2.8 (SD = 6.7). While we centered the efficacy on DukeACT observations, we varied the efficacy of CB on VABS-3 communication subscale score extensively, including 0.

#### Intervention safety

CB infusion in DukeACT was safe, with similar rates of mild and moderate adverse events in the intervention and placebo arms (CB: 4.2% mild, 5.0% moderate; Placebo: 6.6% mild, 4.9% moderate) [8]. There were 4 severe adverse events in the CB group (3.4%) that were all infusion reactions. In the base-case CB strategy, we incorporate a 9.2% probability for mild/moderate adverse events (3.4% for severe) during the first month. Mild/moderate events incur a 0.25 day decrease in QOL and severe events incur a 1-week decrement. We assumed that experiencing a severe adverse event stops the therapy immediately and no intervention benefit is imparted. We assumed no adverse events in the SOC group.

#### Quality of life

Quality of life scores for each simulated participant during ages 2–17 years were derived using a published regression mapping equation (See **Box 1**) that incorporates VABS subscale scores, IQ, and ADOS severity scores to yield an overall quality of life score between -0.36 and 1 [18].

**Box 1. Mapping equation for QOL, ages 2–17 years**

-0.1630 + (0.0037*VABS_Communication) + (0.0046*VABS_DailyLivingSkills)+ (0.0010*VABS_Socialization)—(0.005*ADOS) + (0.024*log(IQ))

When applying the mapping equation, we assumed NVIQ as a proxy for IQ and VABS-3 subscale scores for VABS-II subscale scores. The intervention effect was added to the *VABS_Communication* variable over the first 6 months of the simulation, but the incremental difference between intervention and control groups would last over a lifetime as we did not subsequently subtract the intervention effect after 6 months. All VABS subscore components changed during ages 2–7 consistent with published VABS changes during that period. QOL for ages >17 years was assumed to be equivalent between CB and SOC strategies regardless of VABS-3 score, and was obtained from a study of intellectually able elderly adults with ASD [2]. Therefore, the incremental difference in utilities between an intervention and control group affecting VABS scores would only accrue during ages <17 years. We did not adjust quality of life based on symptom severity or IQ [18, 19].

#### Costs

Yearly costs for children ages 2–17 years included excess healthcare costs, school costs, ASD-specific therapies, and caregiver time for individuals with ASD when compared with demographic and non-ASD-illness adjusted peers, resulting in an estimated cost of $19,199/year [20]. Costs for individuals with ASD and without intellectual disability aged 18+ years were $56,559, and included accommodation costs, employment support costs, medical and non-medical services costs, and caregiver and participant productivity loss costs [3]. It is unlikely that the annual costs for adults with ASD are more than three times those for children, and our basecase adult cost is likely an overestimate [21]. However, in the absence of other published estimates on the societal costs of adult ASD, we include these costs our basecase analysis and perform sensitivity analyses by reducing adult costs by 3x, to align better with the pediatric cost data source. All costs were assumed equivalent regardless of VABS-3 composite score, except for adult productivity loss costs, which were adjusted as detailed below. As such, any reduction in adult ASD medical costs would not affect incremental costs of the CB intervention, but instead impact total costs of both the SOC and CB interventions. All costs were updated to 2019 US dollars using published GDP deflators [22].

The cost of CB infusion in the intervention arm in the base-case analysis was assumed to be $15,000. For reference, previous research cites a $3,000 cost for private umbilical cord blood banking at a storage facility for 20 years, and outpatient infusion costs at $3–4,000 [23–25]. In the base-case analysis, we assumed a one-time infusion, however, given the potential need for up to three subsequent infusions we varied the cost of CB from $8,000–45,000 [6, 26]. Costs of adverse events were incorporated as one-time $2,100 cost for mild/moderate and $11,600 for severe grades, assumed to be similar to outpatient and inpatient managed infusion reactions in older adults [27].

#### Productivity

As IQ explains only a very small share of inter-individual differences in employment and earnings for individuals with ASD without intellectual disability, we assumed VABS-3 communication scores affected employment and productivity levels in our simulated cohort [28–31]. In the base-case analysis, we assumed 60% labor force participation for individuals with a VABS-3>85, 40% with VABS-3 70–85, and 20% with VABS-3 <70 and tested these assumptions in sensitivity analyses, where we lowered productivity differences to 0.

### 2.4 Sensitivity analyses

We performed one-way sensitivity analyses on input parameters to determine their effect on our findings. We combined influential parameters to produce best and worst-case scenarios for cost-effectiveness of the intervention. We also performed structural sensitivity analyses that varied the utility mapping equation and efficacy input data extracted from DukeACT (Appendix 3 in S1 File). To inform future trials, we conducted a two-way sensitivity analysis examining differential CB efficacy and CB cost combinations, on cost-effectiveness findings.

### 2.5 Budget impact analysis

We projected 5-year undiscounted costs from the healthcare payer perspective, comparing incremental costs of the CB strategy over SOC to estimate the budget impact of providing CB to the entire eligible US population of children with ASD and without intellectual disability. We assumed 100% uptake of the intervention to estimate an upper bound of costs. To account for the potential that CB therapy has no significant impact, we additionally projected budgetary losses if a non-efficacious therapy is implemented.

## 3. Results

### 3.1 Model validation

Life expectancy in both the SOC and CB strategies was 66.5 years, consistent with a population-based estimate of 67 years [32]. Mean change in VABS-3 score from ages 2–7 was 6.2, similar to estimates of a change of 6.4 in published developmental models [12, 33]. At 6 months (after the intervention period), the CB strategy had a mean increase in VABS-3 communication subscale score of 2.94 which is consistent with the data imputed from DukeACT. Our model predicted discounted lifetime cost in the SOC strategy of $1,014,000, which is different than a literature-based estimate of $1.43M [3]. The reason for this difference is our use of a different annual cost estimate through ages 2–18 ($19,199 versus an estimated $50–60,000) which we chose due to consistency with other estimates of costs of pediatric autism [20, 34]. However, if similar costs to that estimate are used in our model during ages 2–18, the model projected discounted lifetime cost for SOC is $1.47M, which is consistent with the literature-based estimate.

### 3.2 Clinical results

Assuming the basecase efficacy of CB of 3.0, mean undiscounted QALYs in the SOC and CB strategies were 40.75 and 40.91 QALYs, with the CB imparting a 0.16 QALY increase over a lifetime (**Table 2**). Discounted lifetime QALYs were 16.83 and 16.96 in the SOC and CB strategies.

**Table 2:** Lifetime societal costs and quality-adjusted life years.

Strategy	Lifetime QALYs, undiscounted	Lifetime QALYs, discounted	Lifetime Costs, discounted, $	ICER, $/QALY
**SOC**	40.75	16.83	$1,014,000	-
**CB cost $15,000**	40.91	16.96	$1,028,000	$105,000/QALY
**CB cost $8,000**	-	-	$1,021,000	$51,000/QALY
**CB cost $25,000**	-	-	$1,038,000	$183,000/QALY
**CB cost $35,000**	-	-	$1,048,000	$261,000/QALY
**CB cost $45,000**	-	-	$1,058,000	$338,000/QALY

CB: cord blood, ICER: incremental cost-effectiveness ratio, SOC: Standard of care, QALY: quality-adjusted life year.

Costs and QALYs are discounted at 3%/year for calculation of incremental cost-effectiveness rations.

### 3.3 Costs and cost-effectiveness

Discounted lifetime societal costs in the SOC strategy were $1,014,000 (**Table 2)**. In the CB strategy, with a single $15,000 infusion cost, lifetime societal discounted costs were $1,028,000 yielding an incremental lifetime cost of $14,000 for the CB strategy. At higher infusion costs, lifetime societal discounted costs increased to $1,038,000 with a $25,000 intervention, $1,048,000 with an $35,000 intervention, and $1,058,000 with a $45,000 intervention. When a healthcare payer perspective was taken, lifetime discounted costs were $276,000 for SOC and $292,000 for CB.

Assuming a $15,000 cost for treatment, the CB intervention with an efficacy of 3.0 was borderline cost-effective from a societal perspective, with an incremental cost effectiveness ratio (ICER) of $105,000/QALY (**Table 2**). At CB intervention costs below $15,000, CB crosses below the $100,000/QALY threshold. Cost-effectiveness at a $15,000 intervention cost from a healthcare payer perspective was $121,000/QALY.

### 3.4 Sensitivity analyses

**Fig 1:**
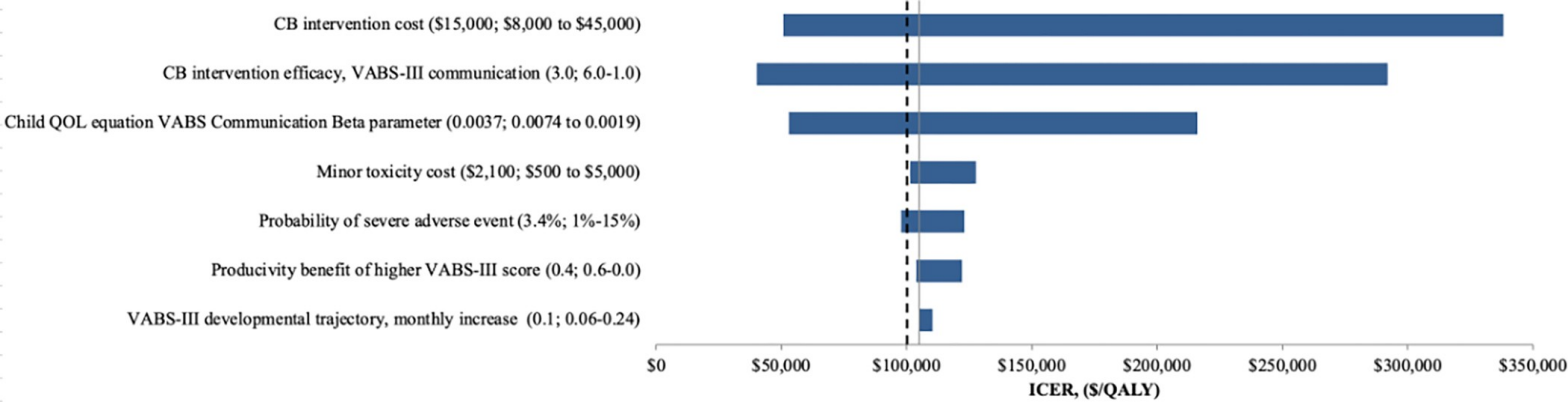
Tornado diagram of cost-effectiveness of one-way sensitivity analyses. This Figure shows the influence of several individual parameters, varied across their plausible ranges, on cost-effectiveness on the CB intervention. Ranges assessed are presented as (base-case value: value that yields lowest ICER—value that yields highest ICER). The four most influential parameters on cost-effectiveness results were CB cost, CB efficacy, child QOL mapping equation beta parameter, and minor toxicity cost. CB: cord blood, ICER: incremental cost-effectiveness ratio, QALY: quality-adjusted life year, QOL: quality of life, VABS-3: Vineland Adaptive Behavior Scale-3.

**Fig 2:**
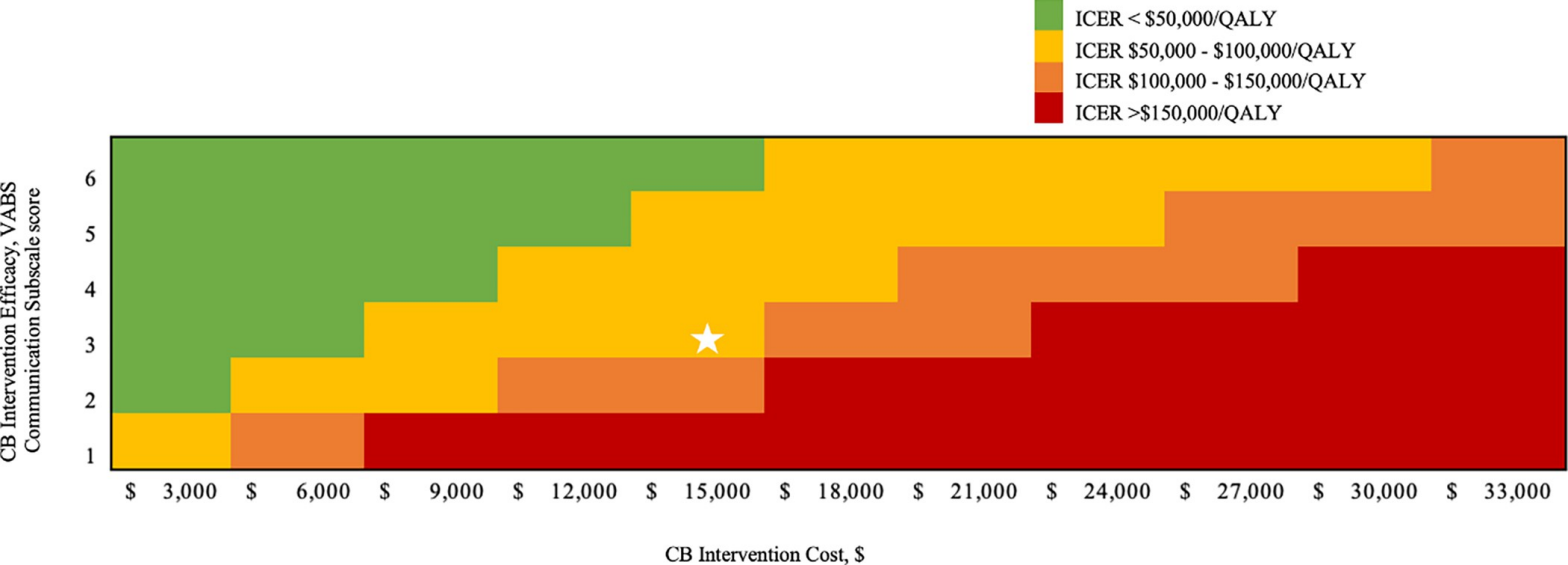
Two-way sensitivity analysis on cord blood intervention efficacy and cost. This figure shows the effect of varying CB intervention efficacy and cost on cost-effectiveness outcomes. CB intervention efficacy on VABS-3 communication subscale score was varied across the Y-axis, from a mean change of 1.0–6.0. CB intervention cost was varied across the X-axis, ranging from $3,000-$33,000. The projected ICER of each combination of efficacy and cost are shown in the Figure and are color coded: green indicates an ICER <$50,000/QALY, yellow an ICER between $50,000–100,000/QALY, orange between $100,000–150,000/QALY, and red >$150,000/QALY (Legend). The white star indicates the projected cost-effectiveness of DukeACT intervention basecase parameters. CB: cord blood, VABS: Vineland Adaptive Behavior Scale, ICER: incremental cost-effectiveness ratio.

Our cost-effectiveness findings were most sensitive to variations in CB intervention cost, CB intervention efficacy, and the child QOL mapping equation VABS communication subscale score beta parameter (**Fig 1**). When only intervention efficacies in VABS-3 communication subscale score greater than the reported minimal clinically important difference in VABS-3 (2.0) were incorporated (all others were 0), the ICER rose to $167,000/QALY. When we reduced adult non-productivity costs by 3x, discounted lifetime societal costs were reduced for both strategies (SOC = $583,000, CB = $597,000) but the ICER did not change. Simultaneously varying CB efficacy from 1.0–6.0, and CB cost from $3,000-$33,000, we found that, in general, intervention costs <$15,000 and efficacies ≥2.0 resulted in ICERs <$150,000/QALY (**Fig 2**). When CB efficacy was 0, the SOC strategy was dominating, i.e., CB was an inefficient use of resources.

To create a best-case scenario, we simultaneously varied CB cost to $8,000, efficacy to a VABS communication increase of 6.0, beta parameter to 0.0074, and productivity benefits to 60%. With these assumptions, the CB intervention ICER lowered to $8,000/QALY. In the worst-case scenario (that may be expected with a waning intervention that requires several additional doses), we varied CB cost to $45,000, efficacy to a VABS-3 communication increase of 1.0, beta parameter to 0.0019, and productivity benefits to 0%, yielding an ICER of $2,387,000/QALY. We acknowledge that a worst-case scenario might also include a CB efficacy of 0, under which CB would be an inefficient use of resources.

### 3.5 Budget impact analysis

The difference in 5-year undiscounted costs from the healthcare payer perspective was $16,000. If the CB intervention were offered to all 247,000 children ages 2–7 years with ASD but without intellectual disability in the United States, the projected 5-year incremental payer cost of CB compared to SOC would be $3.847B [35, 36]. If the efficacy of CB were 0, then this amount would be lost to a non-efficacious intervention. Reduced uptake of CB would lead to a proportionally reduced budget impact estimate, i.e., if only 20% of children ages 2–7 with ASD took up the CB intervention, the budget impact would be reduced by 80% to $0.769B.

## 4. Discussion

In this modeling study we estimated the long-term clinical and economic effects of a potential and novel therapy for children with ASD. We developed and validated a simulation model that incorporates VABS scores to track adaptive behavior development, and costs of ASD. Our results showed that even a modestly effective future intervention that improves adaptive behavior can be cost-effective over a lifetime. CB remained cost-effective (<$100,000/QALY) when the intervention cost was <$15,000. In sensitivity analyses, we found that the cost-effectiveness results were most sensitive to variations in intervention cost, intervention efficacy, and changes in the QOL mapping equation. Of note, this analysis does not project the cost-effectiveness of cord blood therapy as trialed in DukeACT that did not have clinical efficacy, but rather a potential future intervention that is found efficacious in a well-conducted clinical trial.

Our results might be used as a preliminary guide to future clinical trial VABS-3 communication subscale score endpoints that may be cost-effective. We showed that future interventions with costs <$15,000 and efficacies on VABS-3 communication subscale score ≥2.0 might be cost-effective with an ICER<$150,000/QALY. This modeling framework may further be used for value-of-information analysis to help inform study design and sample size for these future trials [37].

We also find that providing such an intervention to every child 2–7 years old with ASD (without intellectual disability) in the US would be costly. Assuming an intervention cost of $15,000, and 100% uptake, the 5-year budget impact on the healthcare sector of providing the intervention was upwards of $3.8B. That said, uptake is likely to be much less than 100% and a one-time CB treatment might be less costly than providing intensive behavioral interventions.

The high cost of the CB intervention simulated in this study must be taken into context with the high costs and healthcare system burden of alternative effective therapies in ASD [34]. Intensive behavioral interventions, such as the Early Start Denver Model (ESDM), are the current gold standard therapies available that improve developmental trajectory in ASD [5]. Cost estimates of ESDM show a $14,000 additional cost in the first 2 years of therapy, but with cost savings over the subsequent post-intervention years due to decreased educational and healthcare resource utilization [38]. As a conservative measure and because these ESDM cost estimates may not be generalizable past the original ESDM research study, we did not decrease childhood costs in our simulation for those receiving the intervention, but it is plausible that similar resource utilization reduction may occur with the CB intervention [4, 38]. Intensive behavioral interventions require highly trained clinicians and significant time investments in implementation in non-academic health centers [38, 39]. It is unlikely that infusion would replace behavioral intervention but rather CB theoretically might augment their efficacy.

We understand that–once an intervention is proven efficacious–there will remain questions about long-term effectiveness relative to upfront costs. In our current volume-based, fee-for-service healthcare reimbursement system, these concerns could lead to payment and coverage challenges and barriers to access. To address these concerns, a decision modeling framework such as ours might inform value-based payment (VBP) models for proven efficacious therapies. Such a payment model would need to align payer evidentiary demands; identify, capture (and link payments to) improved outcomes that matter to participants and payers and incentivizes better long-term evidence.

This is among the first microsimulation models of ASD over the lifetime that incorporates adaptive behavior scores to track developmental trajectories in ASD. Many previously published decision modeling and cost-effectiveness analyses in ASD use IQ strata (i.e. IQ≥70 and IQ<70) to determine future outcomes [40, 41]. While intellectual disability measured by IQ has been shown to have the greatest predictive effect on adult outcomes, such as employment status, it is not useful when predicting outcomes in individuals without intellectual disability [29–31, 40].

The CB intervention efficacy on VABS assumed in our basecase, and factored into QALY calculations, analysis fits well within the range of minimally important clinical difference estimates, which range between 2.01–3.75 [42]. As a conservative measure, we did not incorporate any estimate of caregiver quality of life due to conflicting evidence on the role of adaptive behavior on affecting such caregiver outcomes [43, 44]. We also did not model differences in VABS during adulthood affecting QOL, and their inclusion might further support cost-effectiveness findings [19, 45, 46].

The population simulated in our analysis was based on the participants in DukeACT, however, we note this population is not representative of the general US population [6]. Additionally, our analysis excluded children with intellectual disability (ID), which is a common comorbidity in ASD, and use in this population if unproven would worsen cost-effectiveness. We also recognize the large financial barriers associated with harvesting and storage of autologous cord blood [23]. Publicly available donor cord blood may provide one solution to this problem, or if cord blood storage receives coverage.

Our study has several limitations. First, we recognize the uncertainties in our underlying input data and the assumptions that are inherent in simulation modeling analyses. We presented sensitivity analysis to test these assumptions. Modeling developmental trajectories in ASD, like many neurodevelopmental disorders, requires simplification of individual heterogeneity. In simulating the VABS trajectory, we used literature estimates to simulate individual trajectories yearly from ages 2–7 years [12, 33]. However, we were unable to account for the extent that certain developmental trajectories were associated with better or worse response to the CB intervention due to lack of correlational data. Given lack of currently available data, we unable to project VABS trajectories past year 7 but the modeling framework we developed is readily adaptable to include this data as it is available. Additionally, we assumed an efficacious CB intervention for the purposes of informing future ASD trial design and drug development, assuming clinicians and policymakers would not implement a non-efficacious therapy. There are two placebo-controlled trials of CB in ASD that did not show significant effects on the primary outcome, and as such, our cost-effectiveness results should be considered as theoretical and informing future therapy and clinical trial development [7]. These assumptions were relaxed in sensitivity analyses where we lowered the efficacy of the intervention to 0, and where we reduced the productivity benefits in adulthood to 0.

Second, in the basecase, we incorporated DukeACT 6-month efficacy data to inform our basecase intervention and assumed CB intervention efficacy lasted throughout childhood (for quality of life) and adulthood (for economic productivity). While it is likely that the adaptive behavioral skills acquired 6-months post intervention in DukeACT are not lost, long-term similar follow-up data for the intervention are not available. DukeACT collected 12-month data, however these data were collected remotely and are not comparable to the 6-month data. We addressed this limitation in sensitivity analyses where we lowered the efficacy of the CB intervention to 0, and where we reduced the productivity benefits in adulthood to 0. Third, our total adult ASD costs were an overestimate given inconsistencies in the published literature, however these costs did not affect cost-effectiveness or budget impact estimates as seen in sensitivity analysis [3, 21]. Future research might provide better estimates of the medical and non-medical costs of adult ASD for incorporation in economic analysis. Lastly, this study did not explicitly compare CB with intensive behavioral interventions. Future research comparing CB and intensive behavioral intervention might elucidate the separate, or perhaps even synergistic, effects of each individual intervention, as well as their combination.

Overall, we find that a novel cord blood intervention in ASD with modest efficacy may be cost-effective under certain circumstances. Our projections may guide researchers and policymakers in their consideration of future novel therapeutics in ASD.

## Supporting information

S1 File (DOCX)
