# Supplementary material for: The potential cost-effectiveness of novel cord blood therapies in children with autism spectrum disorder
Source: PLoS One. 2023 Apr 18;18(4):e0282906. doi: 10.1371/journal.pone.0282906 (PMC10112778; doi:10.1371/journal.pone.0282906)
Supplement: S1 File — (DOCX) [file pone.0282906.s001.docx]

**The Potential Cost-Effectiveness of Novel Cord Blood Therapies in Children with Autism Spectrum Disorder**

**Technical Appendix**

Ethan D. Borre, PhD

Evan Myers, MD, MPH

Marianne Hamilton Lopez, PhD, MPA

Joanne Kurtzberg, MD

Beth Shaz, MD

Jesse Troy, PhD

Gillian Sanders Schmidler, PhD

**Appendix 1. Model Flow Diagram**

**Figure S1.**

**
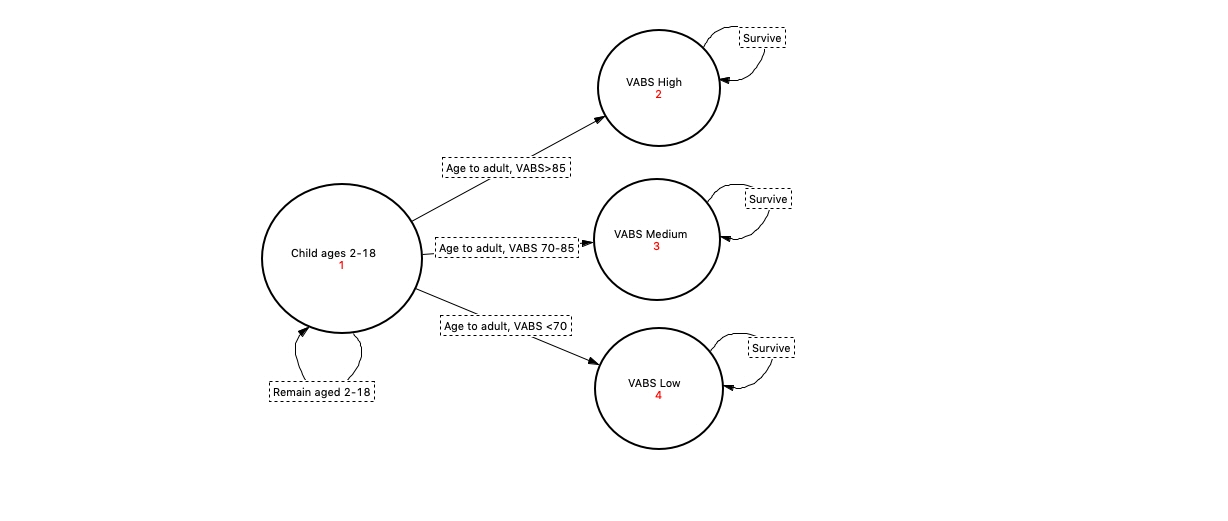
**

**Legend to Figure S1.**

This figure is a model schematic of the Markov health-state transition model developed for this analysis. Exclusive health-states are represented by large circles, transitions are represented by straight arrows, and circular arrows represent remaining in the same health state. Simulated individuals begin the left-most health state (Child Ages 2-18) and remain in that health state until they reach 18.0 years. At age 18.0 years, simulated individuals transition to the adult health states based on their VABS-III at that point in time (Low: VABS <70, Medium: 70-85, and High: >85). Literature-derived effectiveness (QOL) and costs are accrued monthly based on the health state the simulated individual is in (see Methods above). There is a consuming health state, death (not shown), that can be entered from any other health state based on monthly age-, sex-, and autism-specific mortality rates. VABS: Vineland Adaptive Behavior Scale.

**Appendix 2. The distribution of basecase CB efficacy**

In the basecase analysis, we used the mean and standard deviation of the changes in VABS-III communication subscores seen in the placebo and CB intervention arms of DukeACT. Implemented in the modeling software as a normal distribution, we report in the Table below the cumulative distribution function of change scores drawn for SOC and CB that are negative, between 0-2.0, and >2.0. We report around 2.0 as this is a reported minimally clinically important difference in VABS-III. Please see the main manuscript for a sensitivity analysis that demonstrates the impact on cost-effectiveness results of only incorporating drawn efficacies of >2.0.

|  | Worse (negative change) | No Change (0-2.0) | Improved (2.01 and above) |
| --- | --- | --- | --- |
| SOC | 50% | 10% | 40% |
| CB | 35% | 10% | 55% |

**Appendix 3. Structural Sensitivity Analyses**

We conducted 2 sensitivity analysis that differed in their methods of incorporating DukeACT CB intervention efficacy into the Markov microsimulation model. The methods varied either quality of life (QOL) mapping equation that correlated VABS subscores (communication, socialization, and daily living) to quality adjusted life years (QALYs); or the efficacy parameters of VABS and subscores observed in DukeACT. An overview of each method is below.

1. Method 1 (base-case): In the base-case Method 1 we used the QOL mapping equation that correlated VABS *subscores* (communication, socialization, and daily living) to QALYs. The CB efficacy parameters we used were the CB and placebo group-specific communication subscore changes seen in DukeACT, but then the averaged changes across all arms for the socialization and daily living subscores. For Methods 1 and 2, we assume that the mean developmental trajectory of the composite score is divided evenly amongst the three subscore components (their trajectory is highly correlated). This method was chosen as the basecase method because it was felt to be the most conservative method, only incorporating the statistically significant change in communication subscore attained in DukeACT.
2. Method 2: In Method 2 we similarly used the QOL mapping equation that correlated VABS *subscores* (communication, socialization, and daily living) to QALYs However, in this method, we incorporated the CB group and placebo-group specific 6-month changes in all three subscores: communication, socialization, and daily living. This method better accounts for trends in CB benefits to socialization and daily living VABS subscores, but as they were not statistically significantly different in DukeACT we did not select this method as our basecase analysis.
3. Method 3: In Method 3, we used a different QOL mapping equation that related the VABS composite score to QALYs, rather than individual subscores. To extrapolate the effect of the communication subscore benefit seen in DukeACT to the composite score, we weighted the communication subscore benefit by 3 to add to the composite VABS score. This Method was not selected as the base-case for our analysis because DukeACT did not observe a statistically significant different in composite VABS scores between the CB and Placebo arms.

*Adaptive Behavior to QOL Mapping Equation for Methods 1+2*

This is the identical mapping equation used in the basecase analysis.

-0.1630 + (0.0037*VABS_Communication) + (0.0046*VABS_DailyLivingSkills)+ (0.0010*VABS_Socialization) - (0.005*ADOS) + (0.024*log(IQ))

*Method 1 (basecase) efficacy data:*

We used DukeACT Placebo and CB-specific 6-month subscore changes for communication subscore, but the weighted average across all groups for the socialization and daily living subscores. The shading of the cells indicates the arm of the cost-effectiveness analysis that each data point was incorporated into: blue shading = incorporated into SOC arm, green shading = incorporated into CB arm, grey shading = incorporated into SOC and CB arms.

| **Group** | **Socialization Mean Change (SD)** | **Communication Mean Change (SD)** | **Daily Living Mean Change (SD)** |
| --- | --- | --- | --- |
| DukeACT Placebo | - | 0.11 (7.29) | - |
| DukeACT CB | - | 2.96 (7.90) | - |
| DukeACT Placebo+CB | 3.17 (9.17) | 1.97 (7.69) | 2.76 (6.62) |

*Method 2 efficacy data:*

| **Group** | **Socialization Mean Change (SD)** | **Communication Mean Change (SD)** | **Daily Living Mean Change (SD)** |
| --- | --- | --- | --- |
| DukeACT Placebo | 2.09 (8.73) | 0.11 (7.29) | 2.74 (6.08) |
| DukeACT CB | 3.75 (9.40) | 2.96 (7.90) | 2.77 (6.91) |
| DukeACT Placebo+CB | - | - | - |

*Method 3 mapping equation and efficacy data*

Methods 3 used a different mapping equation that incorporated the adaptive behavior composite-score as opposed to the individual subscores.

-0.2438 + (0.0103*VABS_Composite) - (0.0063*ADOS) + (0.0304*log(IQ))

Efficacy Data: We incorporated the VABS communication subscore mean changes from DukeACT, weighted them, and added them to the VABS baseline composite score over 6 months.

| **Group** | **Communication Mean Change (SD)** |
| --- | --- |
| Placebo | 0.11 (7.29) |
| CB Weighted Averages | 2.96 (7.90) |

*Results of the 3 Methods*

| Strategy | Lifetime QALYs, undiscounted | Lifetime QALYs, discounted | Lifetime Costs, $ | ICER, $/QALY |
| --- | --- | --- | --- | --- |
| Method 1: Basecase | | | | |
| SOC | - | 16.85 | 973,000 | - |
| CB cost $15,000 | - | 16.98 | 986,000 | $101,000/QALY |
| Method 2 | | | | |
| SOC | - | 16.68 | $928,000 | - |
| CB cost $15,000 | - | 16.85 | $940,000 | $71,000/QALY |
| Method 3 | | | | |
| SOC | - | 16.27 | $928,000 | - |
| CB cost $15,000 | - | 16.40 | $942,000 | $108,000/QALY |

**Appendix 4. CHEERS Checklist.**

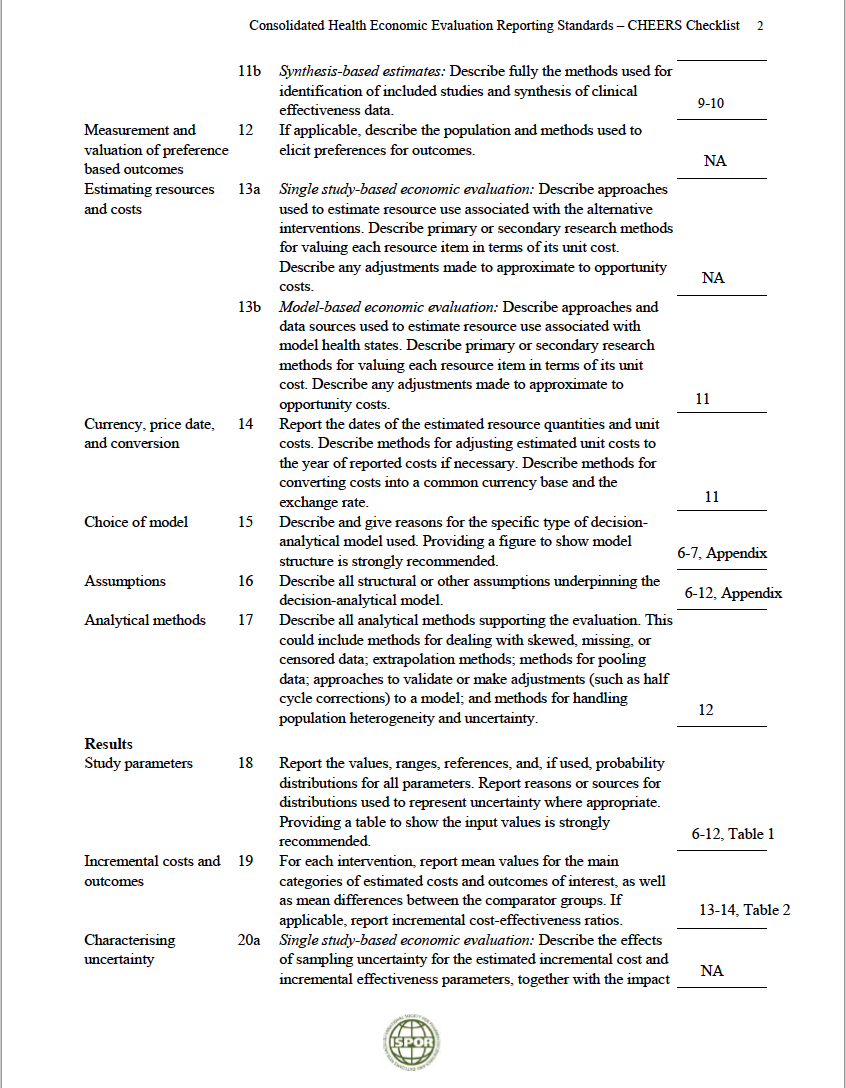


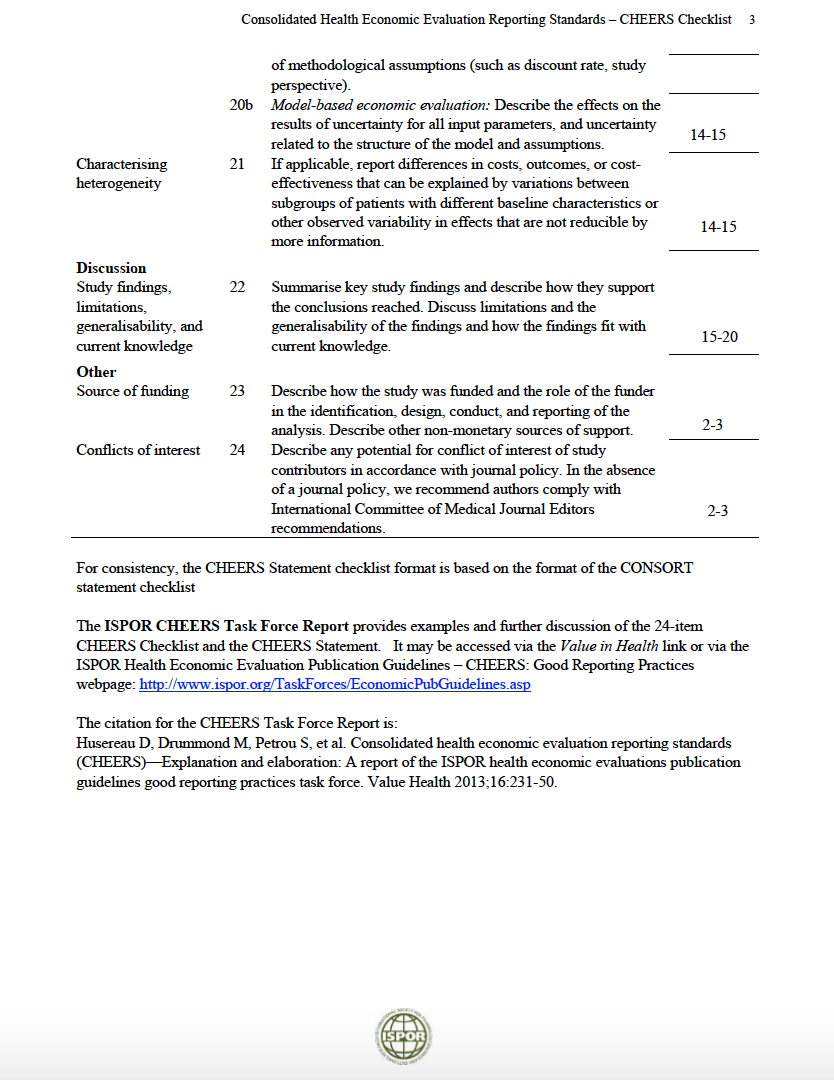


**Appendix 5.** Impact Inventory Detailing Costs Included in the Healthcare Sector and Modified Societal Perspectives

| **Sector** | **Type of impact** | **Included in Healthcare Sector Perspective** | **Included in Modified Societal Perspective** |
| --- | --- | --- | --- |
| **Formal Health Care Sector** | | | |
| Health outcomes (effects) | Longevity Effects | Yes | Yes |
|  | Health-related quality-of-life effects | Yes | Yes |
|  | Other health effects (e.g. Adverse events and secondary transmissions of infections) | Yes | Yes |
| Medical costs | Paid for by third-party payers | Yes | Yes |
|  | Paid for by patients out-of-pocket | No | No |
|  | Future related medical costs (payers and patients) | No | No |
|  | Future unrelated medical costs (payers and patients) | No | No |
| **Informal Health Care Sector** | | | |
| Health | Patient-time costs | - | Yes |
|  | Unpaid caregiver-time costs | - | Yes |
|  | Transportation costs | - | Yes |
| **Non-Health Care Sector** | | | |
| Productivity | Labor market earnings lost | - | Yes |
|  | Cost of unpaid productivity due to illness | - | Yes |
|  | Cost of uncompensated household production | - | Yes |
| Consumption | Future consumption unrelated to health | - | No |
| Social Services | Cost of social services as a part of intervention | - | Yes |
| Legal or Criminal Justice | Number of crimes related to intervention | - | No |
|  | Cost of crimes related to intervention | - | No |
| Education | Impact of intervention on educational achievement of population | - | No |
| Housing | Cost of intervention on home improvements (e.g., removing lead paint) | - | No |
| Environment | Production of toxic waste pollution by intervention | - | No |
| Other (specify) | Other impacts | - | N/A |
